# Revision and Validation of the Mother-Love Absence Scale

**DOI:** 10.3390/bs15101296

**Published:** 2025-09-23

**Authors:** Yanhui Xiang, Xinping Zhou

**Affiliations:** 1 Department of Psychology, Hunan Normal University, Changsha 410081, China; zhouxinping@hunnu.edu.cn; 2 Cognition and Human Behavior Key Laboratory of Hunan Province, Hunan Normal University, Changsha 410081, China

**Keywords:** psychological absence, mother love, confirmatory factor analysis, measurement invariance

## Abstract

The mother’s love is vital for adolescent development, yet there is a lack of specific tools to measure its psychological absence. This study aimed to develop the Mother-Love Absence Scale (MLAS) by revising the Father-Love Absence Scale (FLAS) and verify its reliability and validity among adolescents. Study 1 included 2700 junior and senior high school adolescents. The junior and senior high school samples were each randomly split into two subsamples. One subsample was used for Exploratory Factor Analysis (EFA), while the other was used for Confirmatory Factor Analysis (CFA), internal consistency reliability tests, and cross-gender measurement invariance tests. The results indicated that the factor structure of the MLAS was consistent with that of the FLAS, both comprising four factors: emotional absence, cognitive absence, behavioral absence, and volitional absence. Furthermore, multi-group confirmatory factor analysis verified the gender invariance of the scale. In Study 2, 193 adolescents were surveyed to conduct a CFA and a criterion-related validity analysis. The results of the CFA again demonstrated a good model fit for the four-factor structure. The criterion-related validity analysis indicated that the mother-love absence was negatively connected with parent–child interaction and positively correlated with parental phubbing. Therefore, the revised MLAS has well reliability and validity and can be a reliable instrument for measuring mother-love absence in adolescents.

## 1. Introduction

This study aimed to revise the Mother-Love Absence Scale (MLAS) based on the Father-Love Absence Scale (FLAS) to measure mother-love absence in adolescents across the four dimensions of fundamental psychological qualities: cognitive, emotional, behavioral, and volitional. Following the definition of father-love absence by Xiang and Zhou (2023), mother-love absence is defined as the mother’s absence in a child’s cognitive, emotional, behavioral, and volitional aspects during their development, with an emphasis on this absence being psychological.

For a long time, the mother’s love has played an irreplaceable role in an individual’s development, and considerable evidence suggests that the absence of the mother’s love in early life can have widespread effects on an individual’s cognitive, emotional, volitional, and behavioral development. Specifically, cognitively, maternal support is crucial for early intellectual development (Dunkel et al., 2023), and longitudinal research indicates that maternal sensitivity has lasting impacts on children’s language, executive function, and academic outcomes, both in early and later life (Foley et al., 2025). Emotionally, maternal emotional absence can foster insecure attachment patterns (Bretherton, 1985), and the negative affect generated by maternal antagonism impairs early adolescents’ ability to regulate their emotions (Moilanen & Rambo-Hernandez, 2017). With respect to volition, maternal autonomy support contributes positively to adolescents’ psychological adjustment (Soenens et al., 2007). Behaviorally, authoritarian maternal parenting is more likely to elicit aggressive behaviors in adolescence (Masud et al., 2019), while positive mother–child interactions are shown to promote prosocial behavior (Ferreira et al., 2016).

Although the importance of a mother’s love for an individual’s development has been widely verified, no scale currently exists to directly measure it from cognitive, emotional, behavioral, and volitional perspectives. A review of the literature reveals existing instruments that indirectly measure the mother’s love through constructs such as parenting styles and parents’ emotional attitudes and behaviors toward their children. The Egna Minnen av Barndoms Uppfostran (EMBU) is one such instrument that measures the mothers through parenting styles; it assesses these styles by having participants recall how their parents treated them during their upbringing (Perris et al., 1980), and its revised versions have been widely used in many countries (Arrindell et al., 2005; Z. Li et al., 2012). The EMBU focuses more on measuring the direct, overt behaviors of parenting, such as “My parents praised me” (Z. Li et al., 2012), whereas the MLAS concentrates on the child’s psychological sense of absence, exemplified by items like “To me, I feel like my mother is a stranger.”Additionally, the Parental Acceptance-Rejection Questionnaire (PARQ) is another effective tool for indirectly measuring the mother’s (Khaleque & Rohner, 2002). The PARQ is a survey that people complete themselves, intended to evaluate children’s present impressions and adults’ retrospective recollections of the extent to which they experienced parental acceptance or rejection in their upbringing. Parental acceptance comprises a warmth and affection dimension, while parental rejection includes three dimensions: hostility and aggression, indifference and neglect, and undifferentiated rejection (Rohner & Ali, 2016). Compared to the PARQ, the MLAS adds the dimension of the mother’s influence on the child’s volition, with items such as, “Influenced by my mother, I am more persistent and determined in what I do.” Furthermore, the item phrasing in the MLAS is more culturally attuned to the Chinese context.

The FLAS provides a blueprint for the revision of the MLAS, but whether its theoretical framework and measurement factors are applicable to mother-love absence remains to be verified. The FLAS is an instrument that systematically assesses the phenomenon of the psychological absence of fathers’ love from the perspective of adolescent subjective experience. Based on the theory of the individual’s fundamental psychological qualities, the scale was developed with four core factors: cognitive, emotional, volitional, and behavioral absence, and it has demonstrated good structural validity among Chinese adolescents (Xiang & Zhou, 2023). First, there is a theoretical basis for revising the FLAS into the MLAS. As cognition, emotion, volition, and behavior constitute an individual’s underlying psychological diathesis (Hong, 2004), the influences of the fathers’ love and the mother’s love on a child or adolescent in these four aspects are consistent, with both aiming to help the individual become a well-rounded person in these areas. Second, revising FLAS into MLAS has practical significance. Although the fathers’ love and the mother’s love have consistent influences on an individual’s cognitive, emotional, volitional, and behavioral development, their specific manifestations differ. For example, a mother’s love may promote a child’s social-emotional development, while a father’s love is often related to children’s behavioral problems and mental illnesses (X. Li & Meier, 2017). Therefore, the revision of the MLAS is supported by a theoretical basis and practical significance. Furthermore, the revised scale can directly measure the degree of the mother’s across cognitive, emotional, volitional, and behavioral domains, which will facilitate direct research into the impact of the mother’s love on adolescent development.

Therefore, the current study creates the MLAS by revising the word “father” to “mother” in the FLAS. This direct adaptation is based on the theoretical premise that the fundamental psychological impacts of parental love’s absence are similar for both parents, a premise empirically supported by the results of the present study. The reliability and validity of this new scale will be systematically verified through two studies. Study 1 aims to establish its structural validity and internal consistency reliability, while Study 2 aims to verify its criterion validity by examining the correlations between mother-love absence, parent–child interaction, and parental phubbing.

## 2. Study 1

In Study 1, we predict that the influence of the mother’s love on a child or an adolescent is consistent with that of fathers’ love across the cognitive, emotional, volitional, and behavioral aspects. Therefore, we will use exploratory factor analysis (EFA) and confirmatory factor analysis (CFA) to verify if MLAS shares the same factor structure as the FLAS, and multi-group confirmatory factor analysis (MGCFAs) to assess gender invariance, thereby testing the applicability of the MLAS to adolescents of different genders. At the same time, considering that adolescence is a stage of rapid physical and psychological growth (Steinberg & Morris, 2001), differences exist in the psychophysical characteristics between junior and senior high school students. Therefore, Study 1 will conduct EFA, CFA, and cross-gender measurement invariance tests separately for junior and senior high school groups.

### 2.1. Materials and Methods

#### 2.1.1. Participants and Procedure

We used cluster sampling to administer a questionnaire survey to students from one junior high school and one senior high school in Central China. The initial sample consisted of 2726 participants. Our discard criteria targeted participants who had missing data across all items or provided invalid scores outside the possible range of the scale, resulting in a final sample of 2700 for the subsequent analyses. This included 1261 junior high school students and 1439 senior high school students. The final sample consisted of 1390 male students (51.5%) and 1310 female students (48.5%). The ages of the junior high school students ranged from 11 to 16 years (*M* = 12.97, *SD* = 0.61), while the senior high school students’ ages ranged from 12 to 18 years (*M* = 15.74, *SD* = 0.57). This study randomly split the junior and senior high school samples for EFA (junior high *n* = 631, senior high *n* = 720) and CFA (junior high *n* = 630, senior high *n* = 719).

Before distributing the questionnaire, consent was obtained from school administrators, relevant teachers, and students’ parents. All participants were informed that their participation was voluntary and that they could withdraw at will. Every participant signed an informed consent before completing the questionnaire, and they received appropriate compensation. This research was approved by the ethics committee of the authors’ university on 21 March 2025.

#### 2.1.2. Measure

##### The Mother-Love Absence Scale (MLAS)

The Mother-Love Absence Scale (MLAS) was adapted from the Father-Love Absence Scale (FLAS) by replacing the word “father” with “mother” in each of the original items. The FLAS was developed to assess the extent of father-love absence within the Chinese cultural context (Xiang & Zhou, 2023). The MLAS comprises 18 items across four dimensions: cognitive absence (items 1–6), emotional absence (items 7–11), behavioral absence (items 12–15), and volitional absence (items 16–18), with the full list of items available in Appendix A. Each item is scored on a 5-point scale ranging from 1 (completely inconsistent) to 5 (completely consistent). Higher scores indicate a greater degree of mother-love absence. Items 1–15 are scored directly, while items 16–18 are reverse-scored. In Study 1, the MLAS demonstrated good reliability, with Cronbach’s alpha coefficients of 0.874 for junior high school students and 0.901 for senior high school students.

#### 2.1.3. Data Analyses

The data were analyzed with SPSS Version 26 (IBM Corporation, Armonk, NY, USA) and Mplus Version 8.3 (Muthén & Muthén, Los Angeles, CA, USA). First, an EFA was conducted using SPSS 26.0 to identify the underlying factor structure; then, a CFA was performed using Mplus 8.3 to verify the goodness-of-fit of the factor structure. Subsequently, MGCFAs were performed to test for measurement invariance across genders. Ultimately, Cronbach’s alpha coefficient was computed to assess the internal consistency reliability. For the CFA, model fit was deemed acceptable when the subsequent conditions were satisfied: Comparative Fit Index (CFI) > 0.90, Tucker–Lewis Index (TLI) > 0.90, and root mean squared error of approximation (RMSEA) < 0.08, standardized root mean squared residual (SRMR) < 0.08 (Browne et al., 1993; Kline, 2023). For gender measurement invariance, the alteration in coefficients of fit was used as the criterion, with invariance being supported by ΔCFI ≤ 0.010 and ΔRMSEA ≤ 0.015 (Sass, 2011; Tan et al., 2022).

### 2.2. Results

#### 2.2.1. Item Analysis

First, we used the critical ratio method to validate item discrimination. Participants were ranked based on their total scores on the MLAS and were divided into a high-scoring group (the first 27% of the total score) and a low-scoring group (the last 27% of the total score). An independent samples t-test was then conducted to analyze the differences between the two groups. The results showed that the differences in the scores for each item between these two groups reached a statistically significant level (*p* < 0.001).

Subsequently, we analyzed the item-total correlations using Pearson correlation analysis. For the junior high school sample, the correlations ranged from 0.30 to 0.66, and for the senior high school sample, from 0.38 to 0.76, all of which were highly significant (*p* < 0.001). With the exception of items 16 (r = 0.30), 17 (r = 0.36), and 18 (r = 0.39) in the junior high sample, and item 16 (r = 0.38) in the senior high sample, all other correlations were above 0.40. Although the correlation coefficients for items 16, 17, and 18 were relatively low, they were retained because they are core items for measuring the volitional dimension. This dimension is an essential component of the fundamental psychological qualities theoretical framework. Therefore, to maintain the theoretical structural integrity of the scale, we decided to retain these items.

#### 2.2.2. Exploratory Factor Analysis

The junior and senior high school samples were each randomly split in half. From these, subsamples of 631 junior high and 720 senior high school students were used for EFA. First, the Kaiser–Meyer–Olkin values were 0.894 and 0.899 for the junior and senior high school samples, respectively, which are greater than 0.600. Bartlett’s Tests of Sphericity were also significant for both the junior high (χ^2^ = 4932.685, *p* < 0.001) and senior high (χ^2^ = 7079.526, *p* < 0.001) samples, indicating that the data were suitable for factor analysis (Boateng et al., 2018). Subsequently, principal component analysis with a direct oblimin rotation was used to extract factors with eigenvalues greater than 1. The results revealed a four-factor structure for both samples, accounting for 64.58% and 67.97% of the total variance for the junior and senior high school groups, respectively. Item factor loadings for the junior high sample ranged from 0.43 to 0.90, while loadings for the senior high sample ranged from 0.45 to 0.91. All factor loadings were greater than 0.40, indicating their significance (Boateng et al., 2018). The specific results are presented in Table 1.

#### 2.2.3. Confirmatory Factor Analysis

**Figure 1:**
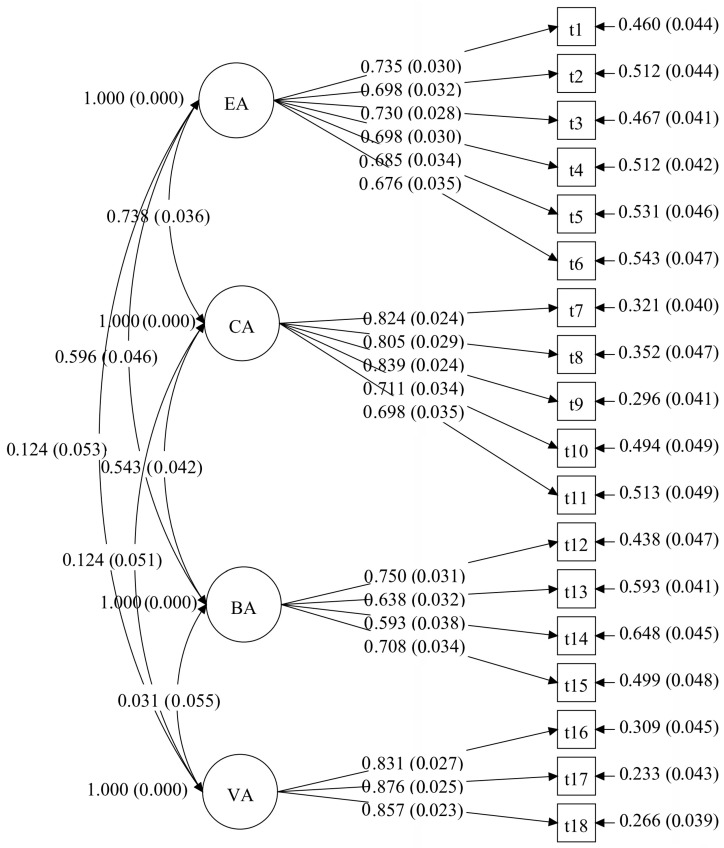
The CFA model of the MLAS for the junior high school group in study 1. Note. EA is emotional absence; CA is cognitive absence; BA is behavioral absence; VA is volitional absence. Model paths show standardized factor loadings with standard errors in parentheses. The curved lines indicate inter-factor correlations.

**Figure 2:**
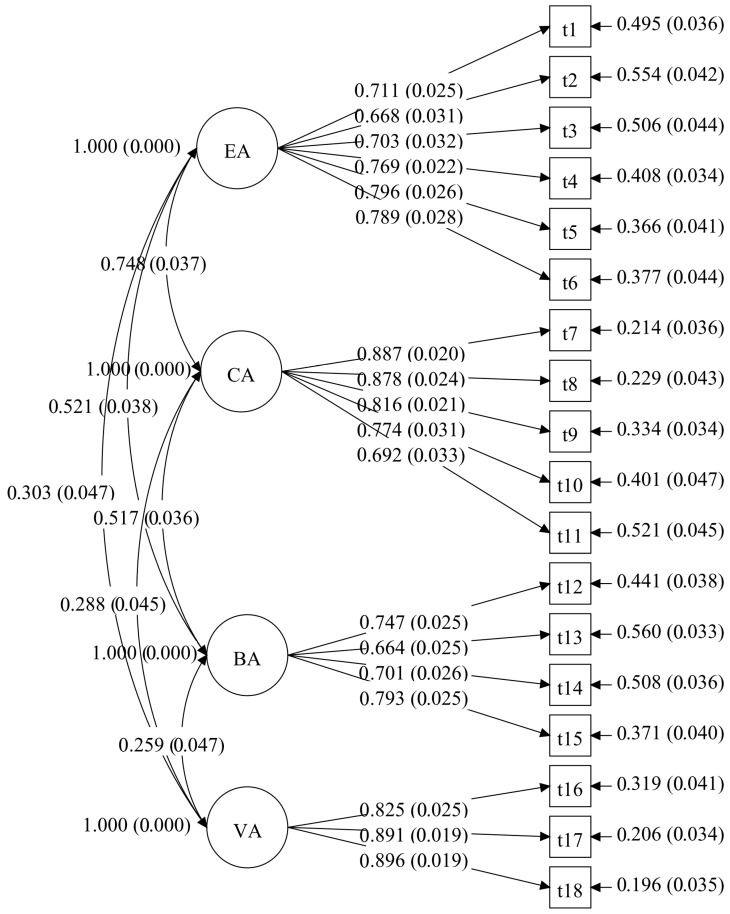
The CFA model of the MLAS for the senior high school group in study 1. Note. EA is emotional absence; CA is cognitive absence; BA is behavioral absence; VA is volitional absence. Model paths show standardized factor loadings with standard errors in parentheses. The curved lines indicate inter-factor correlations.

A CFA was carried out in Mplus 8.3 utilizing maximum likelihood estimation (MLR) to evaluate the structure of factors of the MLAS. (see Figure 1 and Figure 2). For both the junior high and senior high school groups, the analysis revealed an acceptable model fit. For the junior high school group: χ^2^ = 305.296, *df* = 129, CFI = 0.95, RMSEA = 0.05, SRMR = 0.05, TLI = 0.94. For the senior high school group: χ^2^ = 590.470, *df* = 129, CFI = 0.91, RMSEA = 0.07, SRMR = 0.05, TLI = 0.90.

#### 2.2.4. Internal Consistency Reliability

The Cronbach’s alpha coefficients for the total scale were 0.874 and 0.901 for the junior and senior high school groups, respectively, with subscale coefficients ranging from 0.766 to 0.890 for junior high students and 0.815–0.904 for senior high students. The internal consistency reliabilities for the subscales are shown in Table 2.

#### 2.2.5. Measurement Invariance Across Genders

As shown in Table 3, the initial step in the invariance testing procedure was to establish the configural model, which serves as the baseline model for subsequent analyses. The model fit indices for both the junior high group (CFI = 0.944, RMSEA = 0.051, SRMR = 0.055) and the senior high group (CFI = 0.913, RMSEA = 0.073, SRMR = 0.062) met the criteria for configural invariance.

Next, metric invariance was tested by constraining the factor loadings to be equal across groups. The constrained model maintained an adequate fit for both the junior high and senior high school samples. Crucially, the model fit did not significantly deteriorate when compared to the baseline configural model for either the junior high group (ΔCFI = 0.002, ΔRMSEA = −0.002) or the senior high group (ΔCFI = −0.003, ΔRMSEA = −0.001), thus providing support for weak invariance.

Subsequently, based on the metric invariance model, scalar invariance was measured by constraining the item intercepts to be equal across groups. The results again showed good model fit for both groups. In comparison to the metric invariance model, there were no significant differences in fit indices for the junior high group (ΔCFI = −0.004, ΔRMSEA = 0.000) or the senior high group (ΔCFI = −0.003, ΔRMSEA = −0.001), indicating that strong invariance was supported.

Finally, error variance invariance was tested by constraining all error variances to be equal across groups and examining whether each item had the same level of measurement error between groups (Doré et al., 2017). The results for the junior high group (ΔCFI = −0.010, ΔRMSEA = 0.003) and the senior high group (ΔCFI = 0.003, ΔRMSEA = −0.003) indicated that further constraining the error variances did not lead to a significant change in the model.

The above results indicate that the perception of mother-love absence is consistent across genders for adolescents in both junior and senior high school.

## 3. Study 2

Study 1 verified that the factor structure of the MLAS is consistent with that of the FLAS, and that it possesses good reliability and cross-gender invariance among Chinese adolescents. In Study 2, after confirming the factor structure in the current sample, we further verified the criterion validity of the MLAS. Mother-love absence measures the psychological absence of a mother in a child’s cognitive, emotional, behavioral, and volitional life. This psychological absence can be perceived not only directly by the adolescent but also indirectly through the mother’s behaviors during the adolescent’s development. Therefore, we will verify the criterion-related validity of the MLAS from two aspects: internal psychological experience and external behaviors.

Parent–child interaction includes three dimensions: intimacy and safety; tolerance and support; and understanding and empathy. Intimacy and safety relate to the harmony, emotional experience, and satisfaction during interactions; tolerance and support relate to trust, forgiveness, and parental cooperation; understanding and empathy relate to attitudes of knowing and understanding during interactions (Xiao, 2020). The Parent–Child Interaction Scale explores whether a child experiences psychological feelings such as safety, trust, autonomy, and empathy within the parent–child relationship through specific interactive contexts. Studies have consistently found that supportive and affectionate parent–child relationships are essential for an individual’s flourishing social-emotional growth and the formation of secure attachment. (McCoby, 1983; Sroufe, 2005). High-quality parent–child interaction can be seen as an indicator of sufficient mother’s love. If a child reports high-quality parent–child interaction, their perceived level of mother-love absence should be lower. Therefore, we used parent–child interaction as the criterion for the internal psychological aspect and expected its score to be negatively correlated with the MLAS score.

A detrimental behavior that has become common in the digital age, parental phubbing describes a parent’s absorption with their smartphone at the expense of interacting with their child (Zhang et al., 2023). Evidence indicates that this behavior negatively impacts the parent–child bond and may also be associated with the emergence of psychological difficulties and problematic internet use among adolescents (Komanchuk et al., 2023). The more frequently adolescents perceive parental phubbing, the more likely they are to feel neglected, thus experiencing a stronger sense of mother-love absence. Therefore, we used parental phubbing as the criterion for the external behavioral aspect and predicted that the MLAS score would be positively correlated with the parental phubbing score.

In summary, Study 2 used parent–child interaction as the internal psychological criterion and parental phubbing as the external behavioral criterion to verify the criterion-related validity of the MLAS.

### 3.1. Materials and Methods

#### 3.1.1. Participants and Procedure

Study 2 was conducted with 193 junior high school students (43.5% female) from a junior high school in Central China. The participating adolescents were between 10 and 14 years old (*M* = 12.73, *SD* = 0.53). As part of a psychological quality diary survey organized by the school, participants completed the MLAS, a questionnaire on parent–child relationship status, and the Parental Phubbing Scale.

Before distributing the questionnaire, consent was obtained from school administrators, relevant teachers, and students’ parents. All participants were informed that their participation was voluntary and that they could withdraw at will. Every participant signed an informed consent before completing the questionnaire, and they received appropriate compensation. This study was approved by the research ethics committee of the authors’ university on 21 March 2025.

#### 3.1.2. Measure

##### Parent–Child Interaction

Parent–child interaction was measured using the Parent–Child Relationship Status Questionnaire developed by Xiao (2020). The questionnaire consists of three dimensions (intimacy and safety; tolerance and support; understanding and empathy). The questionnaire assesses the parent–child relationship with 17 items (e.g., “I am willing to proactively tell my parents about things that happen and how I feel when I am outside”). The questionnaire uses a 5-point Likert scale (1 = strongly disagree, 5 = strongly agree). In the current study, the Cronbach’s alpha coefficient for this scale was 0.947.

##### Parental Phubbing

The Parental Phubbing Scale, which is a revised version of the Partner Phubbing Scale (Roberts & David, 2016), is designed to evaluate adolescents’ perceptions of their parents’ phubbing behaviors (Wang et al., 2020). The scale contains nine items (e.g., “When I eat with my parents, they look at their mobile phones”), rated on a 5-point scale (1 = Never, 5 = Always). In the current study, the Cronbach’s alpha coefficient for this scale was 0.777.

#### 3.1.3. Data Analyses

Data processing and statistical computations were carried out with the aid of SPSS 26.0 and Mplus 8.3. We conducted an a priori power analysis for our CFA based on the RMSEA test of model fit (MacCallum et al., 1996). Using an online power calculator (Preacher & Coffman, 2006), we determined that a minimum sample of 111 participants was required to achieve 80% power (α = 0.05, *df* = 129) to distinguish between close (RMSEA ≤ 0.05) and mediocre (RMSEA = 0.08) model fit. Our achieved sample of 193 participants exceeds this requirement, indicating sufficient statistical power for the analysis. With the adequacy of the sample size confirmed, we proceeded with the main data analyses for Study 2. First, a CFA was performed using Mplus 8.3 to confirm that the factor structure verified in Study 1 was also applicable to the current sample. Subsequently, a Pearson correlation analysis was conducted using SPSS 26.0 to examine the criterion-related validity.

### 3.2. Results

#### 3.2.1. Confirmatory Factor Analysis

**Figure 3:**
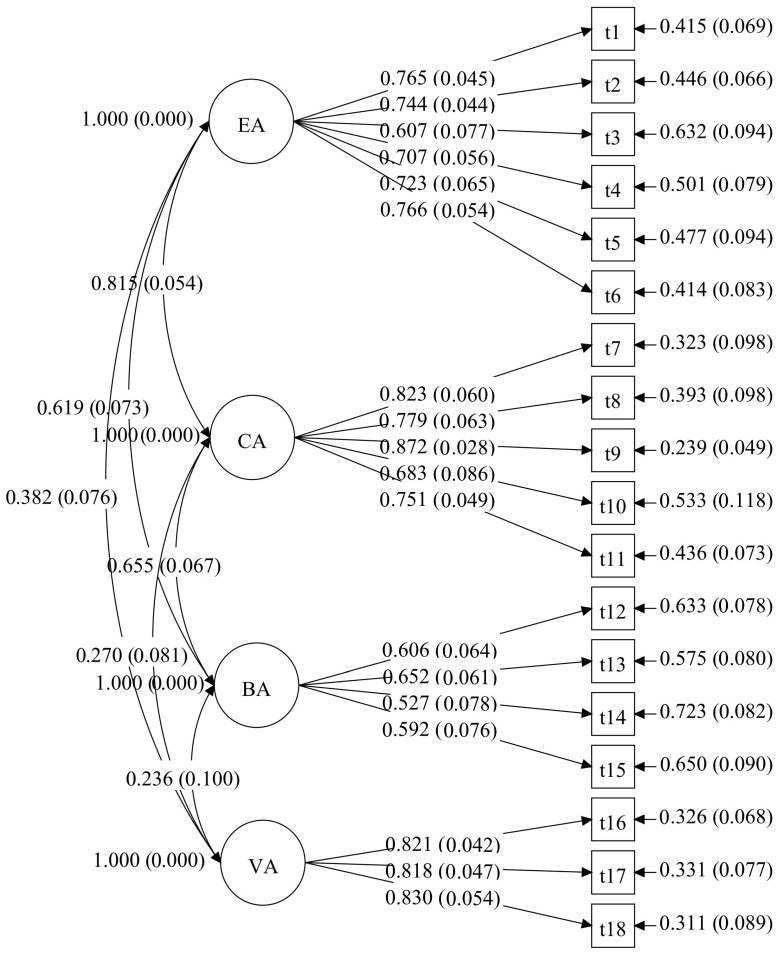
The CFA model of the MLAS for the junior high school group in study 2. Note. EA is emotional absence; CA is cognitive absence; BA is behavioral absence; VA is volitional absence. Model paths show standardized factor loadings with standard errors in parentheses. The curved lines indicate inter-factor correlations.

A CFA was conducted in Mplus 8.3 using MLR to confirm the factor structure of the MLAS (see Figure 3). The analysis revealed that the model provided a good fit to the data: χ^2^/*df* = 1.620, CFI = 0.93, RMSEA = 0.06, SRMR = 0.05, and TLI = 0.92.

#### 3.2.2. Criterion-Related Validity

The criterion-related validity of the MLAS was assessed in the current study from the aspects of internal psychological feelings and external behavioral manifestations. We used the three key components of the Parent–Child Relationship Status Questionnaire (intimacy and safety; tolerance and support; and understanding and empathy) as the criterion for internal psychological feelings, and the Parental Phubbing Scale as the criterion representing external behavioral manifestations. As shown in Table 4, overall, mother-love absence was negatively correlated with parent–child interaction and positively correlated with parental phubbing. Specifically, the cognitive, emotional, behavioral, and volitional absence factors of mother-love absence were all negatively correlated with the three subdimensions of parent–child interaction and were positively correlated with parental phubbing (with the exception of volitional absence).

## 4. General Discussion

The mother’s love is a key factor in an individual’s development, yet few scales directly measure an individual’s perceived degree of the mother’s love. Therefore, this study aimed to develop and validate the Mother-Love Absence Scale (MLAS) for adolescents, and the findings provide strong support for its use. A key finding from Study 1 was the confirmation that the four-factor framework of love absence—comprising cognitive, emotional, volitional, and behavioral dimensions—is as applicable to mothers as it is to fathers. This theoretical transferability suggests that these dimensions may represent a core structure in how adolescents perceive parental love, regardless of the parent’s gender. Furthermore, the establishment of measurement invariance across genders demonstrates that the MLAS is a stable and unbiased tool for meaningful group comparisons, which is crucial for both research and clinical practice.

The consistent measurement results for junior and senior high school students on the MLAS demonstrate the cross-stage stability of the factors measured by the scale. The scale is constructed based on the theory of four fundamental psychological qualities and these psychological needs are present throughout the adolescent developmental period. Both junior high students in early adolescence and senior high students in mid-to-late adolescence are in a critical period of self-identity development and require maternal emotional support (Sartor & Youniss, 2002). Moreover, adolescence is a critical period for the maturation of the prefrontal cortex (Dumontheil, 2016). The absence of a mother’s love during this stage can negatively impact the advancement of executive functions and hinder the formation of volitional qualities, such as self-regulation (Moilanen & Rambo-Hernandez, 2017) and delayed gratification (Kidd et al., 2013). At the behavioral level, both junior and senior high school students who lack the warm and sufficient mother’s love may exhibit externalizing problems such as aggression and risk-taking behaviors (Fearon et al., 2010).

In Study 2, the results showed that the total scale and subscales of the MLAS were negatively correlated with the three dimensions representing parent–child interaction: intimacy and safety (r = −0.543), tolerance and support (r = −0.411), and understanding and empathy (r = −0.469). Additionally, the scales were positively correlated with the negative behavior of parental phubbing (r = 0.268). This indicates that the more severe the mother-love absence perceived by adolescents, the poorer the quality of parent–child interaction they report and the more parental phubbing they perceive. These findings confirm that the MLAS score is not an isolated perception but is meaningfully connected to the broader quality of the parent–child relationship, lending support to earlier work on these topics (Liu, 2003; Xie & Xie, 2020). In conclusion, these results demonstrate that the MLAS has good criterion-related validity and can effectively reflect adolescents’ true feelings within the parent–child relationship.

The results collected from Study 1 and Study 2 illustrate that the revised MLAS is a measurement tool with a clear structure and good reliability and validity. The revised scale provides an effective method for directly measuring a mother’s love from the perspective of fundamental psychological qualities in China. Furthermore, the concise and efficient MLAS can help clinicians in practical applications to assess the perceived state of mother-love absence in adolescents and to conduct targeted family interventions.

Although the current study successfully revised the MLAS, certain limitations exist that also point to important directions for future research. First, this study relied on self-report instruments, which may render the findings prone to social desirability bias. Second, the parent–child interaction scale used for criterion validation has not been widely substantiated in international literature. Therefore, the findings regarding the MLAS’s criterion-related validity should be considered preliminary, and future research should validate the MLAS against more established, internationally recognized measures. Additionally, the study only examined the internal consistency reliability of the MLAS; future research could also test its test–retest reliability. Last, the research sample was confined to Chinese teens. To enhance the scale’s ecological validity and generalizability, future research should explore its application among adults, individuals from other nations, and adolescents from alternative family structures (e.g., single-parent households or families with same-sex parents).

## 5. Conclusions

The outcomes substantiate the reliability and validity of the MLAS, establishing it as a psychometrically sound tool. Furthermore, it is well-suited for the Chinese cultural context, providing an effective means to measure the subjective experience of mother-love absence among adolescents.

## Figures and Tables

**Table 1:** Results of Exploratory Factor Analysis for Junior and Senior High School Samples.

Items	Factor Loading for Junior High School				Factor Loading for Senior High School			
	Factor1	Factor 2	Factor 3	Factor 4	Factor1	Factor 2	Factor 3	Factor 4
1	0.77				0.82			
2	0.82				0.83			
3	0.61				0.68			
4	0.73				0.74			
5	0.70				0.71			
6	0.74				0.66			
7				0.86				0.80
8				0.90				0.83
9				0.82				0.66
10				0.72				0.84
11				0.43				0.45
12			0.81				0.82	
13			0.76				0.78	
14			0.73				0.76	
15			0.58				0.72	
16		0.87				0.89		
17		0.90				0.91		
18		0.88				0.90		
The eigenvalue	6.26	2.34	1.64	1.38	6.91	2.23	1.73	1.37
Explain the total variance (%)	34.79	13.01	9.11	7.68	38.36	12.39	9.58	7.63
Cumulative interpretation rate (%)	34.79	47.80	56.91	64.58	38.36	50.75	60.34	67.97

**Table 2:** Internal consistency reliability of the mother-love absence scale for junior and senior high school groups.

	EA	CA	BA	VA
Junior high school	0.854	0.877	0.766	0.890
Senior high school	0.875	0.897	0.815	0.904

Note. EA is emotional absence; CA is cognitive absence; BA is behavioral absence; VA is volitional absence.

**Table 3:** Fit Indices for Measurement Invariance Test Across Genders.

Model	χ^2^	*df*	CFI	RMSEA	SRMR	△CFI	△RMSEA
Junior high school students							
M1	468.693	258	0.944	0.051	0.055		
M2	476.558	272	0.946	0.049	0.057	0.002	−0.002
M3	504.719	286	0.942	0.049	0.057	−0.004	0.000
M4	565.110	304	0.932	0.052	0.061	−0.010	0.003
Senior high school students							
M1	746.032	258	0.913	0.073	0.062		
M2	776.958	272	0.910	0.072	0.065	−0.003	−0.001
M3	802.582	286	0.907	0.071	0.064	−0.003	−0.001
M4	806.788	304	0.910	0.068	0.063	0.003	−0.003

Note. M1 is configural invariance model; M2 is metric invariance model; M3 is scalar invariance model; M4 is error variance invariance model.

**Table 4:** Criterion-related validity of the MLAS.

	EA	CA	BA	VA	MLAS
Parent–Child Interaction–Intimacy and Safety	−0.522 ***	−0.424 ***	−0.381 ***	−0.286 ***	−0.543 ***
Parent–Child Interaction–Tolerance and Support	−0.392 ***	−0.273 ***	−0.239 **	−0.333 ***	−0.411 ***
Parent–Child Interaction–Understanding and Empathy	−0.486 ***	−0.326 ***	−0.300 ***	−0.276 ***	−0.469 ***
Parental Phubbing Scale	0.266 ***	0.225 **	0.168 *	0.137	0.268 ***

Note. EA is emotional absence; CA is cognitive absence; BA is behavioral absence; VA is volitional absence; MLAS is mother-love absence scale. *** *p* < 0.001; ** *p* < 0.01; * *p* < 0.05.

## Data Availability

The data in this study are available from the corresponding authors upon reasonable request.

## Appendix A. Mother-Love Absence Scale (MLAS)

Instructions: Please answer the following questions truthfully based on your family experience and place a check mark (√) next to the option that best applies to you.

(1 = Completely inconsistent; 2 = Mostly inconsistent; 3 = Neutral; 4 = Mostly consistent; 5 = Completely consistent)

1. My mother rarely considers things from my perspective.	1	2	3	4	5
2. My mother always thinks my ideas are childish.	1	2	3	4	5
3. I often feel that I am treated unfairly by my mother.	1	2	3	4	5
4. It is difficult for me to get what I want from my mother.	1	2	3	4	5
5. When I receive honors, my mother rarely shows recognition and praise to me.	1	2	3	4	5
6. My mother is rarely proud of me.	1	2	3	4	5
7. To me, I feel that my mother is like a stranger.	1	2	3	4	5
8. I feel that my mother is not important in my life.	1	2	3	4	5
9. I feel that there is an strange sense of distance between my mother and me.	1	2	3	4	5
10. I feel that my mother is far less important than my father in my life.	1	2	3	4	5
11. I feel that communicating with my mother would be an unsettling thing.	1	2	3	4	5
12. My mother rarely picks me up from school.	1	2	3	4	5
13. My mother has almost never read me a bedtime story.	1	2	3	4	5
14. My mother has almost never attended my parent-teacher conferences.	1	2	3	4	5
15. My mother rarely plays with me.	1	2	3	4	5
16. Influenced by my mother, I am decisive and not sloppy in doing things.	1	2	3	4	5
17. Influenced by my mother, I adhere to my own opinions and do not follow others blindly.	1	2	3	4	5
18. Influenced by my mother, I have more perseverance and willpower in doing things.	1	2	3	4	5
Note. The background color for the final items serves as a visual cue to ensure participants’ attention and prevent accidental omission.					
